# Supporting Option B+ scale up and strengthening the prevention of mother-to-child transmission cascade in central Malawi: results from a serial cross-sectional study

**DOI:** 10.1186/s12879-015-1065-y

**Published:** 2015-08-12

**Authors:** Michael E. Herce, Tiwonge Mtande, Frank Chimbwandira, Innocent Mofolo, Christine K. Chingondole, Nora E. Rosenberg, Kathy E. Lancaster, Esmie Kamanga, Jacqueline Chinkonde, Wiza Kumwenda, Gerald Tegha, Mina C. Hosseinipour, Irving F. Hoffman, Francis E. Martinson, Eva Stein, Charles M. van der Horst

**Affiliations:** Tidziwe Centre, UNC Project—Malawi, Private Bag A-104, Lilongwe, Malawi; University of North Carolina at Chapel Hill, 130 Mason Farm Rd. (Bioinformatics), CB# 7030, Chapel Hill, NC 27599-7030 USA; HIV Unit, Ministry of Health, Government of the Republic of Malawi, P.O. Box 30377, Lilongwe 3, Malawi; UNICEF Malawi, P.O. Box 30375, Lilongwe 3, Malawi

## Abstract

**Background:**

We established Safeguard the Family (STF) to support Ministry of Health (MoH) scale-up of universal antiretroviral therapy (ART) for HIV-infected pregnant and breastfeeding women (Option B+) and to strengthen the prevention of mother-to-child transmission (PMTCT) cascade from HIV testing and counseling (HTC) through maternal ART provision and post-delivery early infant HIV diagnosis (EID). To these ends, we implemented the following interventions in 5 districts: 1) health worker training and mentorship; 2) couples’ HTC and male partner involvement; 3) women’s psychosocial support groups; and 4) health and laboratory system strengthening for EID.

**Methods:**

We conducted a serial cross-sectional study using facility-level quarterly (Q) program data and individual-level infant HIV-1 DNA PCR data to evaluate STF performance on PMTCT indicators for project years (Y) 1 (April—December 2011) through 3 (January—December 2013), and compared these results to national averages.

**Results:**

Facility-level uptake of HTC, ART, infant nevirapine prophylaxis, and infant DNA PCR testing increased significantly from quarterly baselines of 66 % (n/N = 32,433/48,804), 23 % (n/N = 442/1,958), 1 % (n/N = 10/1,958), and 52 % (n/N = 1,385/2,644) to 87 % (n/N = 39,458/45,324), 96 % (n/N = 2,046/2,121), 100 % (n/N = 2,121/2,121), and 62 % (n/N = 1,462/2,340), respectively, by project end (*all p* < 0.001). Quarterly HTC, ART, and infant nevirapine prophylaxis uptake outperformed national averages over years 2–3. While transitioning EID laboratory services to MoH, STF provided first-time HIV-1 DNA PCR testing for 2,226 of 11,261 HIV-exposed infants (20 %) tested in the MoH EID program in STF districts from program inception (Y2) through Y3. Of these, 78 (3.5 %) tested HIV-positive. Among infants with complete documentation (n = 608), median age at first testing decreased from 112 days (interquartile range, IQR: 57–198) in Y2 to 76 days (IQR: 46–152) in Y3 (p < 0.001). During Y3 (only year with national data for comparison), non-significantly fewer exposed infants tested HIV-positive (3.6 %) at first testing in STF districts than nationally (4.1 %) (*p* = 0.4).

**Conclusions:**

STF interventions, integrated within the MoH Option B+ program, achieved favorable HTC, maternal ART, infant prophylaxis, and EID services uptake, and a low proportion of infants found HIV-infected at first DNA PCR testing. Continued investments are needed to strengthen the PMTCT cascade, particularly around EID.

## Background

Malawi is a low-income sub-Saharan Africa (SSA) country heavily burdened by HIV/AIDS, with an adult HIV prevalence of 10.3 % [1]. The Malawi epidemic disproportionately affects women who comprise 59 % of prevalent cases [1]. Consequently, 68,000 neonates are exposed to HIV and 11,000 infants become newly HIV-infected each year, adding to the 100,000 prevalent cases of pediatric HIV/AIDS nationally [2]. Without timely HIV diagnosis, treatment, and care, half of these children will die before their second birthday [3].

Prevention of mother-to-child transmission (PMTCT) interventions are the most effective way to halt HIV acquisition among infants and children in SSA [4]. As PMTCT interventions, including improved HIV testing strategies, maternal combination antiretroviral therapy (ART), and infant nevirapine prophylaxis have become more widely available, marked reductions in mother-to-child HIV transmission (MTCT) throughout the perinatal period have been observed—from a baseline of 20-45 % over a decade ago to less than 5 % under research conditions in the ART era [4–6]. Such reductions may be realized when HIV prevention and treatment interventions are optimized for HIV-infected mothers and their HIV-exposed infants at each step of the PMTCT cascade—from HIV testing and counseling (HTC) and maternal ART initiation through infant nevirapine prophylaxis provision, early infant HIV diagnosis (EID), and maternal-infant follow-up until breastfeeding cessation [7, 8].

Under real-world conditions in resource-limited settings a variety of factors attenuate the effectiveness of PMTCT interventions, including resource limitations, inadequate laboratory capacity, health system inefficiencies, and client loss-to-follow-up [9, 10]. Predictive modeling using data from South Africa, Uganda, and Malawi have estimated that the risk of MTCT remains high in real-world settings, approaching 20 % in programs using short-course ART [11]. In Malawi, as recently as 2010, fewer than half of all HIV-infected pregnant women received any PMTCT intervention, and as many as 14 % of exposed infants were found HIV-infected by early DNA PCR testing [12, 13].

In response to these challenges, in September 2011 the Malawi Ministry of Health (MoH) introduced the pioneering “Option B+” strategy of “test and treat,” lifelong combination ART for all HIV-infected pregnant and breastfeeding women [14, 15]. In only its first year, the MoH reported a dramatic increase in the number of HIV-infected pregnant and breastfeeding women accessing ART for their own health and to reduce MTCT. Indeed, the number of pregnant and breastfeeding women initiating ART increased 748 % with the introduction of Option B+ compared to baseline, resulting in greater population-level ART coverage for HIV-infected women [14, 16]. However, despite these impressive gains, client refusal of ART and dropout from the PMTCT cascade threaten the effectiveness of Option B+ in Malawi. As many as 15 % and 17 % of Option B+ clients, respectively, either do not start ART or become lost to follow-up within 6 months of ART initiation [10, 17]. Such early client drop-out from the PMTCT cascade has deleterious “downstream” effects, as inefficiencies in antenatal PMTCT service delivery manifest as obstacles to diagnosis, treatment, and care further along the cascade, ultimately resulting in higher MTCT risk [11].

For over a decade, the University of North Carolina Project—Malawi (UNC) has provided services and conducted research to strengthen the PMTCT cascade in Malawi’s capital, Lilongwe [18–24]. With MoH support and in response to our funder’s request to translate evidence into programmatic action, from April 2011 through December 2013 UNC expanded its PMTCT program beyond Lilongwe to include five districts in central Malawi (Lilongwe, Dedza, Ntcheu, Mchinji, and Dowa districts), creating the Safeguard the Family (STF) project.

**Fig. 1:**
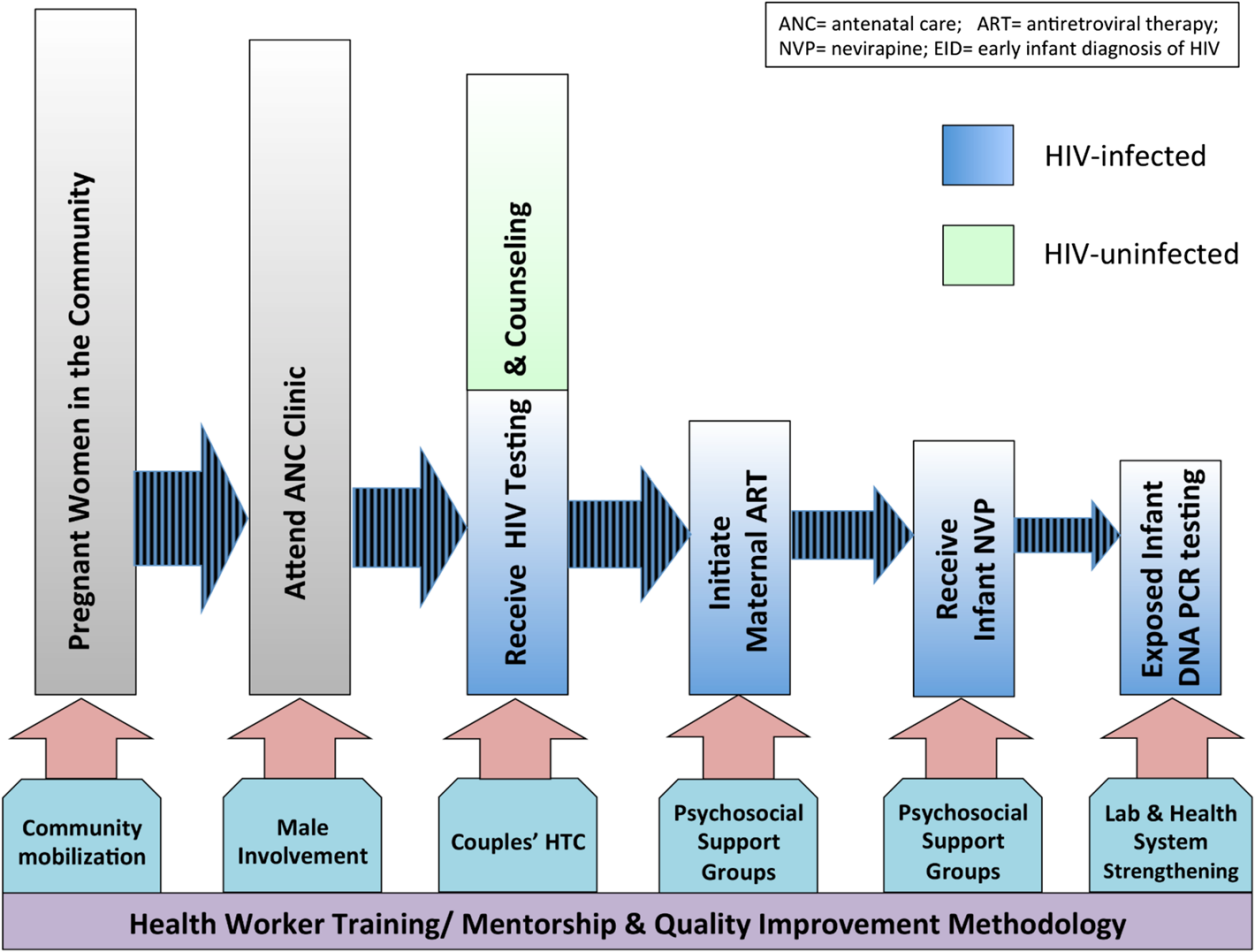
Technical Approach. Safeguard the Family approach to addressing gaps along the antenatal and early postnatal prevention of mother-to-child transmission of HIV cascade

STF had two main objectives: 1) reduce MTCT in the project catchment area; and 2) increase access to high-quality HIV services, including HTC, maternal combination ART (via Option B+), infant nevirapine prophylaxis, and early infant HIV-1 DNA PCR testing, for pregnant women and their HIV-exposed infants during the antenatal and immediate postnatal periods (Fig. 1). In this study, we aim to assess the impact of STF interventions by evaluating STF project outcomes over time and compared to national averages, and by estimating HIV prevalence among HIV-exposed infants at first HIV-1 DNA PCR testing. We also describe the STF implementation model and discuss promising strategies to mitigate barriers to service delivery along the early PMTCT cascade in SSA.

## Methods

### Ethics statement

The National Health Sciences Research Committee of Malawi and the Institutional Review Board of the University of North Carolina, USA, granted ethical approval for all project evaluation elements described herein without requiring patient consent given the retrospective use of de-identified, routinely collected programmatic and clinical data.

### Malawi National PMTCT Program overview

Since 2002, the MoH has offered free PMTCT services, including HTC and anti-retroviral medications, to HIV-infected pregnant women as part of the National ART Program. From 2007 through August 2011—a period encompassing the first 5 months of STF—the National PMTCT Program provided AZT-combination prophylaxis as the standard of care. This prophylactic regimen included, for mothers, daily AZT (beginning in the 28th week of pregnancy) followed by single-dose nevirapine (SD-NVP) plus AZT/3TC at labor onset and a post-delivery 1-week AZT/3TC tail, and, for infants, SD-NVP plus 1 to 4 weeks of AZT syrup (with infant AZT course determined by the duration of maternal prophylaxis) [25]. With the introduction of Option B+ in September 2011, the recommended PMTCT regimen changed to universal, life-long, fixed-dose combination efavirenz, lamivudine, and tenofovir for mothers, and 6 weeks of nevirapine prophylaxis for infants [26].

### Malawi MoH Option B+ program overview

The MoH Option B+ program takes a public-health approach to PMTCT, and provides the following services as the national standard of care: 1) integrated opt-out HTC and ART services in all ANC clinics, with same-day ART availability for all HIV-infected pregnant women; 2) repeat HTC for women testing negative more than 3 months prior to returning for any health service, including antenatal and obstetrical care; 3) nevirapine syrup provided to HIV-infected mothers during antenatal care (ANC) for infant prophylaxis; and 4) provision of a one-pill, once-per-day combination ART regimen.[14] The MoH Option B+ program encompasses EID services. The MoH EID program provides HIV-exposed infants with early registration and longitudinal follow-up in the MoH HIV Care Clinic (HCC), co-trimoxazole prophylaxis, and qualitative HIV-1 DNA PCR testing using dried blood spot (DBS) technology. The national EID guidelines in place during the STF project period recommended that infant DNA PCR testing be performed as soon as possible after 6 weeks of age [26]. Repeat DNA PCR testing was not routinely performed during the project period, except in cases of overdue or invalid results, or early breastfeeding cessation (typically between infant ages of 7 to 12 months).

### STF project overview

To achieve our project objectives and complement the standard of care provided by the MoH Option B+ program, STF implemented the following interventions, each grounded in quality improvement (QI) methodology and informed by nearly a decade’s worth of operational research and situation analyses conducted by UNC in Lilongwe: 1) training and mentorship of MoH health workers to facilitate rapid scale up and high-quality implementation of Option B+, EID, and other PMTCT services; 2) promoting couples’ HTC and male partner involvement in ANC clinic to improve the PMTCT care-seeking environment; 3) establishing women’s community-based psychosocial support groups to reduce individual barriers to PMTCT service utilization; and 4) strengthening health and laboratory systems to facilitate early infant DNA PCR testing (Fig. 1) [18–24]. During project year (Y) 1 (April–December 2011), we recruited STF project personnel, including technical officers based full-time in each STF district, trained MoH health workers, and collected baseline data. During project years 2 (January–December 2012) and 3 (January–December 2013), we implemented STF interventions to include *all* 136 government health facilities located in our 5-district catchment area.

### STF catchment area and population

**Fig. 2:**
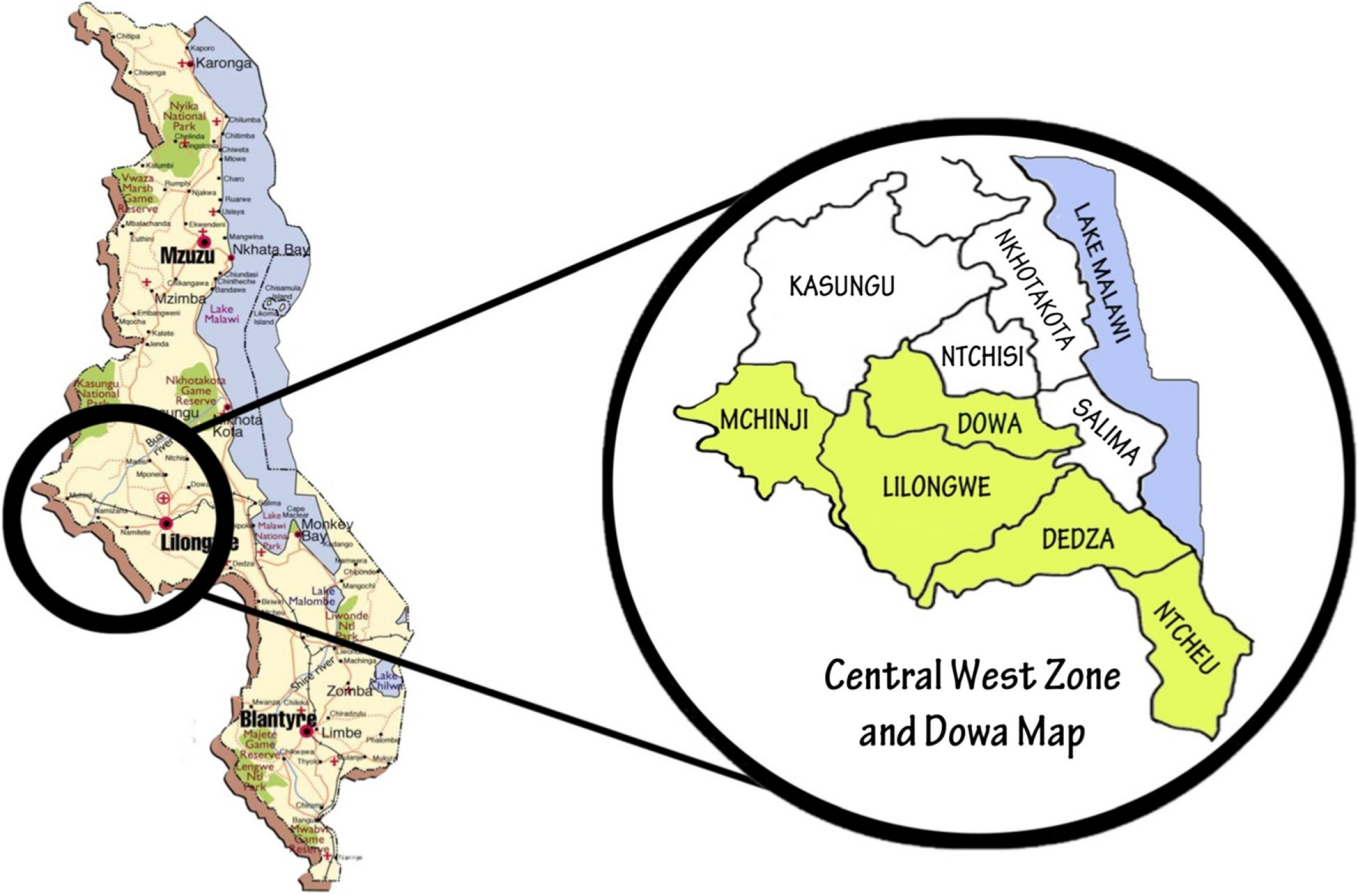
Safeguard the Family catchment area. The 5 districts within the Republic of Malawi served by Safeguard the Family (Malawi country map source: http://www.mappery.com/Malawi-Map-2)

STF supported all government health facilities in 5 districts of central Malawi (comprising the Central West Zone and Dowa district), reaching a total catchment population of 4,420,401 people in 2011 (Fig. 2) [27]. Disaggregated by district, this population included 671,137, 641,895, 511,792, and 513,865 people in rural Dedza, Dowa, Mchinji, and Ntcheu districts, respectively, and 2,081,712 people in urban and peri-urban Lilongwe [27]. On average, 51 % and 38 %, respectively, of the population in rural STF districts and Lilongwe live on less than $0.50 per day [28]. Based on Malawi National Statistics Office data, there were an estimated 208,413 live births in this population in 2011 [27, 29]. In our catchment area and nationally, an estimated 92 % of reproductive-age women attend at least one ANC visit [30].

### Quality improvement (QI) methodology

STF employed QI methodology adapted from an approach successfully used to scale up ART and improve PMTCT services in South Africa [31]. STF technical and monitoring & evaluation (M&E) officers received training and longitudinal mentorship on basic QI methods, such as implementing Plan-Do-Study-Act (*i.e.* PDSA) cycles [32]. Using these methods, STF personnel conducted collaborative, facility-level QI activities with front-line MoH health workers focused on the antenatal and early postnatal PMTCT cascade. Field-based STF staff met with MoH health workers at least once monthly to facilitate: 1) identification of implementation gaps at each step in the cascade; 2) participatory compilation of proposed solutions to address identified gaps; 3) iterative application and assessment of solution effectiveness through routine M&E; 4) identification of local health worker “focal persons” to sustain facility-level quality improvement; and 5) diffusion of promising solutions through district-wide joint mentorship visits with MoH staff.

### Health worker training and mentorship

We trained 980 MoH frontline health workers, including 580 health workers on the MoH Integrated HIV Guidelines (encompassing Option B+), 333 on EID, and 62 on use of point-of-care (POC) CD4 count testing (for male partners of women receiving couples’ HTC). We also provided data management training to 25 MoH health information management officers and relevant program coordinators, based at the district health offices, who worked with facility staff to compile and synthesize routine reporting data. To enhance and reinforce training, we conducted 2893 mentorship and QI visits from year 2 quarter (Q) 4 through year 3 Q4. In year 2 Q4, we facilitated formation of joint MoH-STF mentorship teams in each STF-supported district to build MoH health worker capacity. Each team included key PMTCT-focused coordinators and technical staff from the respective MoH district health offices. Mentorship teams visited each health facility in their respective districts to provide clinical mentorship and systems-based guidance to address logistical, patient flow, and supply chain management challenges related to the introduction and scale-up of Option B+. Mentorship teams planned visits to occur with decreasing intensity, providing intensive monthly mentorship before transitioning to less intensive quarterly mentorship when facilities achieved pre-specified MoH benchmarks. These visits complemented MoH quarterly supportive supervision performed as part of the national standard of care. STF and MoH personnel used a mentorship checklist to ensure standardization of mentorship activities and performance evaluations during visits. Importantly, the checklist helped identify missing HIV commodities required for optimal service delivery. STF responded to missing HIV commodities in our catchment area by providing emergency supplies of HIV test kits (Alere Determine™ HIV-1/2, Waltham, Massachusetts, USA) and generic co-trimoxazole and infant nevirapine syrup beginning in year 2 Q2 to alleviate stock outs.

### Promoting couples’ HTC and male partner involvement

To improve the PMTCT care-seeking environment, we implemented a couples’ HTC and male partner involvement intervention. This intervention encompassed: 1) 220 community mobilization talks and dramatizations to sensitize and educate community members about the benefits of early ANC, male partner involvement, couples’ HTC, and ART for individual health and PMTCT; 2) fast-tracking women in the HTC cue who presented to ANC clinic with a male partner; 3) renovating infrastructure at 9 busy ANC clinics to create dedicated HTC space; and 4) providing same-day, POC CD4 count testing to male partners testing HIV-positive at 16 hospitals and clinics to promote early linkage to care and ART initiation [33]. To support our same-day male-partner POC CD4 testing activity, we equipped 16 health facilities with the PIMA™ CD4 Analyzer (Alere, Waltham, Massachusetts, USA) and trained front-line nurses, Health Surveillance Assistants (HSAs), and HTC counselors on its use [33].

### Women’s psychosocial support groups

We established 36 community-based women’s psychosocial support groups in underserved rural areas in our catchment area, each one linked to a nearby health center. A nurse or HSA from the health center, or a peer health educator from the community, led each group. An average of 20 HIV-infected pregnant and breastfeeding mothers joined each support group on a voluntary basis, after being sensitized to their existence during group health talks given in ANC or ART clinics, or through individual HIV post-test counseling sessions. Psychosocial support groups met monthly, providing a safe forum for HIV-infected mothers to share their experiences and discuss issues surrounding HIV-related stigma and discrimination, HIV-status disclosure, and ART adherence. Support group meetings also served as a venue for health workers to provide health education and communication around breastfeeding, infant vaccination, ART and HCC follow-up for mothers and infants, postnatal EID services, and safe breastfeeding weaning. To incentivize participation and support safe breastfeeding weaning, STF provided 2 kg of vitamin-fortified soya-based porridge to these mothers at each group meeting [23].

### Strengthening health and laboratory systems in support of EID

While national laboratory capacity for the MoH EID program was still being built (year 2 Q1 through year 3 Q4), STF (through the UNC laboratory in Lilongwe) performed HIV-1 DNA PCR testing for HIV-exposed infants receiving health services in rural Dedza, Mchinji, and Dowa districts. During this interim period, STF also strengthened ancillary MoH systems in these underserved districts to enable DBS specimen transport to the Kamuzu Central Hospital (KCH) campus in Lilongwe (site of both the KCH and UNC laboratories) and reporting of DNA PCR test results back to referring health facilities. Concurrently, UNC laboratory mentors, with U.S. Centers for Disease Control and Prevention support, helped build MoH laboratory capacity through pre-service training and in-service staff mentorship on qualitative DNA PCR testing at KCH reference laboratory. All qualitative DNA PCR testing was performed using the Amplicor™ diagnostic platform (Roche Diagnostics, Indianapolis, Indiana, USA).

In all project districts, STF trained MoH health workers on the screening, enrollment, and testing of HIV-exposed infants. Trained HTC counselors collected DBS samples for EID testing per MoH guidelines [26]. STF staff mentored health workers on exposed-infant identification, DBS sample collection, and proper HIV-1 DNA PCR test result documentation and communication to mothers.

### Study design

We conducted a serial cross-sectional study to evaluate STF performance on outcomes of interest at quarterly intervals, and compared STF performance to national averages reported quarterly by the MoH for STF years 1 through 3 [34–44]. We compiled anonymized, cross-sectional *facility-level* data to ascertain project outcomes each quarter (Q). We employed a similar cross-sectional approach to analyze *individual-level* HIV-1 DNA PCR testing outcomes for HIV-exposed infants in the STF catchment area.

### Data collection

We collected facility-level data during quarterly field visits, and entered the composite de-identified data into Microsoft Excel (Redmond, Washington, USA). We abstracted data from routine MoH and STF data-recording tools, including an electronic data-recording system used by MoH at each district hospital as well as paper registers and patient treatment cards available in each health center to enable longitudinal patient care and program M&E. We estimated HTC uptake using data abstracted from MoH ANC registers; Option B+ uptake using MoH ANC registers; couples’ HTC uptake using MoH HTC registers; and EID provision using MoH HCC and EID registers augmented by UNC laboratory information management system (LIMS) data. We used de-identified HIV-1 DNA PCR test results abstracted from the UNC LIMS to perform analyses in HIV-exposed infants.

### Analytical approach

Our outcomes of interest were aligned with national PMTCT indicators, and included quarterly assessment of: 1) the proportion of pregnant women undergoing HTC during ANC (*i.e.* the number of pregnant women with a new HTC test result documented in ANC clinic / the total number of pregnant women presenting to ANC clinic without a prior documented positive HIV test result); 2) the proportion of pregnant women who underwent HTC together with a male partner (*i.e.* the number of pregnant women documented to have undergone HTC with a male partner in ANC clinic / the total number of pregnant women documented to have received HTC in ANC clinic); 3) the proportion of HIV-infected pregnant women receiving combination ART during ANC (*i.e.* the number of HIV-infected pregnant women documented to have received combination ART during ANC / the total number of pregnant women newly enrolled in ANC clinic with either newly documented HIV infection or known prior HIV infection); 4) the proportion of HIV-infected pregnant women provided with nevirapine syrup in ANC clinic (*i.e.* the number of HIV-infected women with documented receipt of infant nevirapine syrup in ANC clinic / the total number of HIV-infected pregnant women registered in ANC clinic); and 5) the proportion of HIV-exposed infants who underwent HIV-1 DNA PCR testing (*i.e.* the number of HIV-exposed infants who underwent documented DBS sample collection for HIV-1 DNA PCR testing/the estimated total number of HIV-infected pregnant women presenting to ANC clinic). For this denominator, we used ANC HIV prevalence data to estimate the number of HIV-infected pregnant women among those not receiving HTC, and added this to the number of documented HIV-infected women registered in ANC clinic; this approach provided a more conservative estimate of EID testing uptake. Outcome indicators estimated *facility-level* utilization of HTC, couples’ HTC, maternal ART, infant nevirapine prophylaxis, and HIV-1 DNA PCR testing, respectively. For facility-level analyses, the sample size was determined by the number of pregnant women, HIV-infected pregnant women, and HIV-exposed infants accessing health services in the STF catchment area during the study period. For our *individual-level* analyses of HIV-exposed infant data, we aimed to: 1) estimate the prevalence of HIV-1 infection among HIV-exposed infants receiving *first-time* DNA PCR testing; 2) evaluate individual-level baseline infant demographic and clinical factors associated with age at first HIV-1 DNA PCR testing; and 3) evaluate individual-level baseline demographic and clinical factors associated with *approximate* average HIV-1 DNA PCR test result turnaround time.We approximated test turnaround time using the following definition: time interval in days from the date of HIV-1 DNA PCR test performance to the date when mothers were notified of their infant’s test result. For individual-level analyses, the sample size was determined by the number of HIV-exposed infants provided first-time HIV-1 DNA PCR testing by STF during the study period.

### Data analyses

We present descriptive statistics for all facility- and individual-level outcomes, including frequencies, percentages, and 95 % confidence intervals for categorical variables, and mean, median, and measures of dispersion for continuous variables. We compared proportions for facility-level PMTCT outcome indicators at baseline and end-of-project using the two-sample test of proportions. We compared median approximate test turnaround time and infant age at first HIV-1 DNA PCR testing using the Wilcoxon Rank-Sum test. We restricted our analyses to individuals with complete data for all variables required to conduct a particular analysis and, in the case of indicator calculations, to individuals with a documented outcome event. All statistical analyses were performed using SAS version 9.2 (Cary, NC, USA) and STATA version 12.1 (College Station, TX, USA).

## Results

### Overview

We first report facility-level outcome indicators for 513,098 newly registered ANC attendees (both HIV-positive and HIV-negative pregnant women) served by STF over its 33-month project lifespan, including 141,519, 189,915, and 177,709 pregnant women accessing facility-based ANC services in years 1 through 3, respectively. We then present our individual-level analyses of HIV-1 DNA PCR test results for HIV-exposed infants.

### HIV testing and counseling

**Fig. 3:**
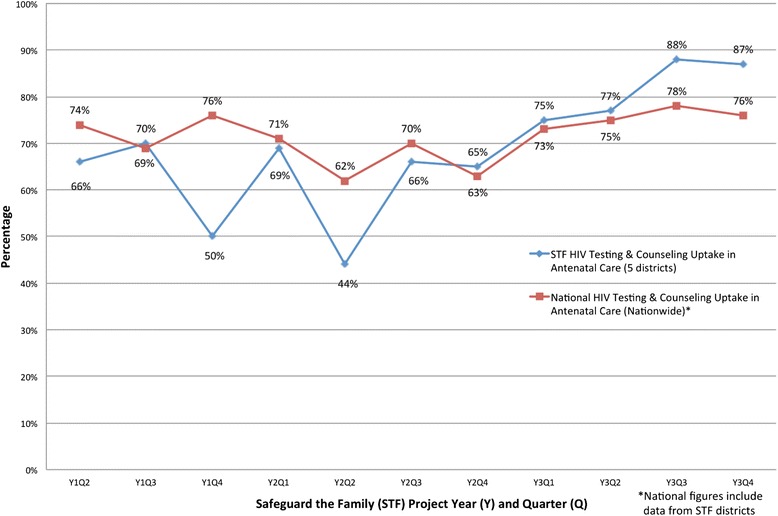
HIV testing & counseling (HTC) uptake. Quarterly comparison of antenatal clinic HTC uptake in the Safeguard the Family catchment area compared to the national average for project years 1 to 3 (April 2011 through December 2013)

Over the entire project period, 1.6 % (n/N = 8,441/513,098) of ANC attendees were known to be HIV-infected at the time of ANC enrollment. Quarterly uptake of HTC among ANC attendees without a documented positive HIV test result in the preceding 3 months increased significantly from a baseline of 66 % (n/N = 32,433/48,804) to 87 % (n/N = 39,458/45,324) by project end (*p* < 0.001) (Fig. 3). Conversely, the annual proportion of eligible pregnant women who did not receive HTC for any reason during their first ANC visit decreased significantly from 37 % in year 1 (n/N = 51,332/139,259) to 17 % in year 3 (n/N = 28,927/174,492) (p < 0.001). After below average performance in year 1, STF-supported districts slightly outperformed the quarterly national average (mean difference: 0.4 %) for HTC uptake in ANC clinic during project years 2 and 3 (Fig. 3).

**Fig. 4:**
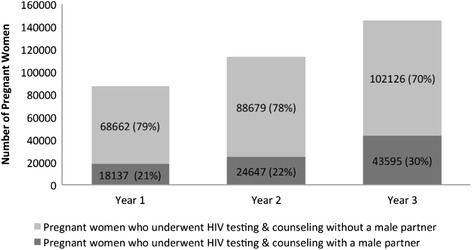
Couples’ HIV testing & counseling (HTC) uptake. Annual uptake of couples’ HTC in the Safeguard the Family catchment area for project years 1 to 3 (April 2011 through December 2013)

In project year 1, 21 % (n/N =18,137/86,799) of pregnant women received facility-based couples’ HTC. In project year 3, 30 % (n/N = 43,595/145,721) of pregnant women accessing HTC in ANC underwent HTC accompanied by a male partner (Fig. 4). The proportion of pregnant women who underwent facility-based couples’ HTC increased significantly from project start (Y1) to end (Y3) (*p* < 0.001).

The prevalence of documented HIV infection among pregnant women who underwent HTC in ANC clinic decreased significantly from the start to end of STF—from a baseline of 6.4 % (95 % confidence interval, CI: 6.3–6.6 %; n/N = 5,725/89,059) in year 1 and 6.4 % (95 % CI: 6.2–6.5 %; n/N = 7,429/116,290) in year 2 to 5.7 % in year 3 (95 % CI: 5.6–5.8 %; n/N = 8,448/148,938) (*p* < 0.001).

### Combination ART in ANC

**Fig. 5:**
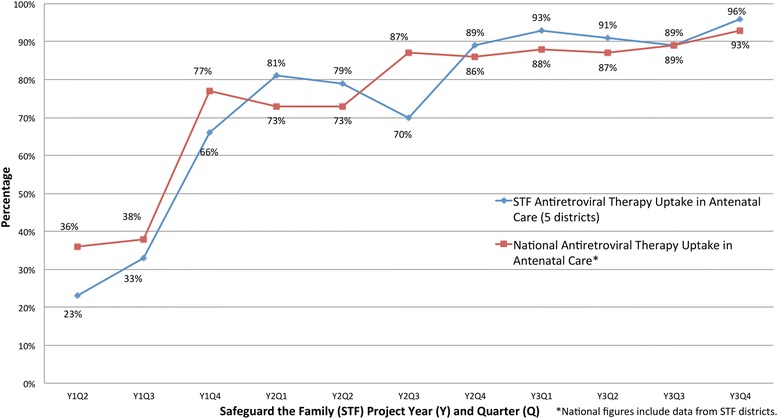
Option B+ uptake. Quarterly comparison of maternal combination antiretroviral therapy / Option B+ uptake among HIV-infected pregnant women presenting to antenatal care clinics in the Safeguard the Family catchment area compared to the national average for project years 1 to 3 (April 2011 through December 2013)

Prior to Option B+ (in Y1 Q2), combination ART quarterly uptake among HIV-infected pregnant women in the STF catchment area was 23 % (n/N = 442/1,958). Following STF health worker trainings that enabled Option B+ roll out to 82 % of health facilities in our catchment area by year 1 end, quarterly ART uptake among HIV-infected pregnant women presenting to ANC increased significantly to 66 % (n/N = 1,057/1,600) by Y1 Q4 (p < 0.001) and 96 % (n/N = 2,046/2,121) by Y3 Q4 (*p* < 0.001), compared to the pre-B+ baseline (Fig. 5). Over the project lifespan, mean quarterly ART uptake in STF-supported districts (74 %) mirrored the national average (75 %). During project years 2 and 3, however, STF-supported districts outperformed the quarterly national average (mean difference: 1.6 %) (Fig. 5).

### Infant nevirapine prophylaxis

**Fig. 6:**
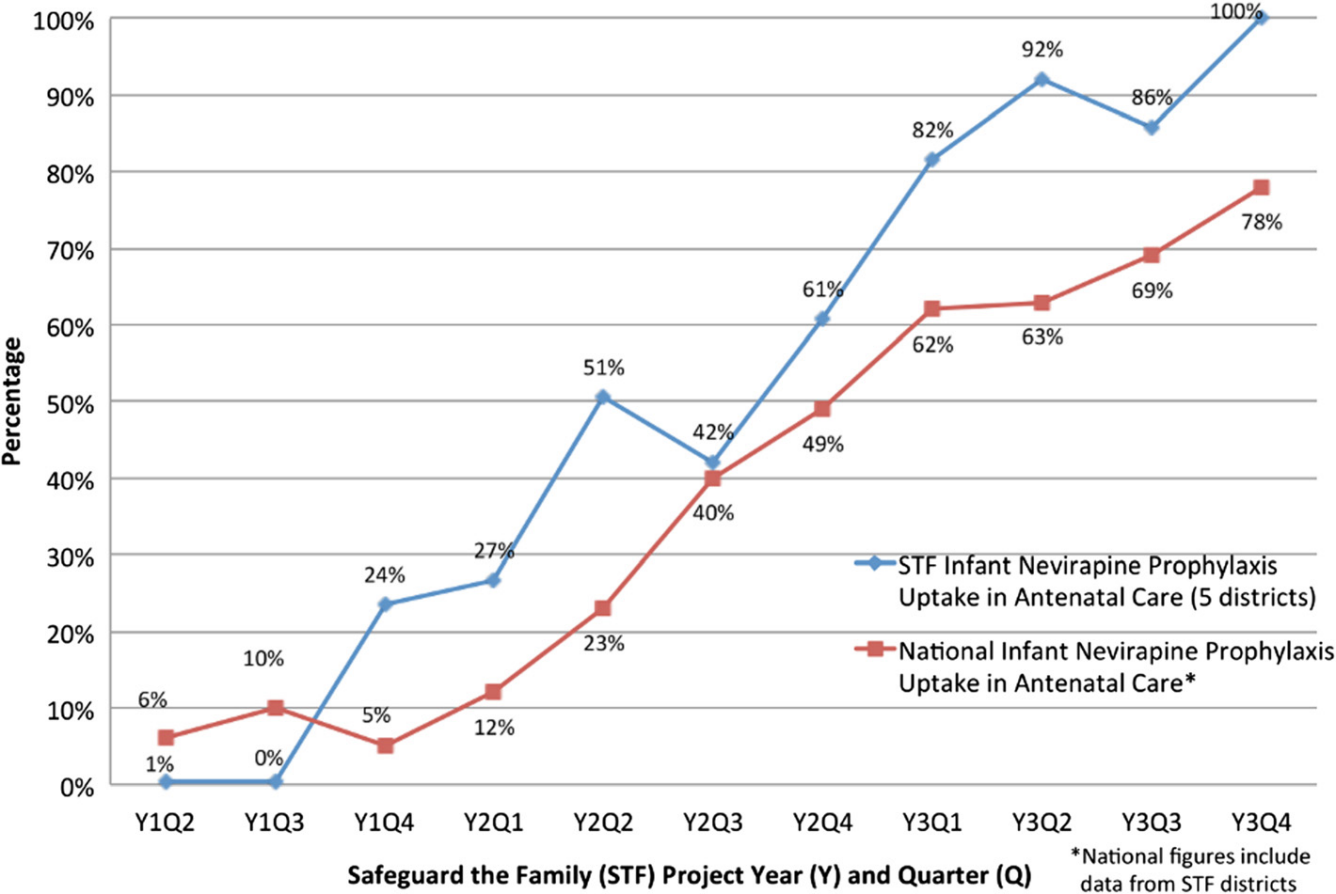
Infant nevirapine prophylaxis uptake. Quarterly comparison of infant nevirapine prophylaxis uptake among HIV-infected pregnant women presenting to antenatal care clinics in the Safeguard the Family catchment area compared to the national average for project years 1 to 3 (April 2011 through December 2013)

Provision of infant nevirapine prophylaxis to mothers in ANC clinic improved each quarter over the project lifespan (Fig. 6). At baseline (Y1 Q2), 1 % (n/N = 10/1,958) of HIV-infected pregnant women presenting quarterly to ANC received nevirapine syrup for infant HIV prophylaxis (Fig. 6). By project end, this had increased significantly to 100 % (n/N = 2,121/2,121) (*p* < 0.001). During years 2 and 3, STF outperformed the quarterly national average by a mean difference of 18 %.

### Early infant diagnosis of HIV infection

The proportion of HIV-exposed infants who had a DBS sample collected in a quarter for first-time HIV-1 DNA PCR testing in STF-supported districts increased significantly from a Y2 Q1 baseline of 52 % (n/N = 1,385/2,644) to 62 % (n/N = 1,462/2,340) by Y3 Q4 (*p* < 0.001). By project year, uptake increased significantly from 46 % (n/N = 5,488/11,916) in year 2 (the first full year that the MoH EID program was implemented in STF districts) to 57 % (n/N = 5,773/10,089) in year 3 (*p* < 0.001).

STF provided interim first-time HIV-1 DNA PCR testing for 2,226 of the 11,261 HIV-exposed infants (20 %) who underwent DBS sample collection in the MoH EID program in our catchment area from program inception (Y2 Q1) through the end of year 3. Of the 2,226 total HIV-exposed infants we tested, 78 were found to be DNA PCR positive for HIV-1 (3.5 %). During year 3 (the only year with national EID data available for comparison), a non-significantly lower proportion of HIV-exposed infants (3.6 %) tested HIV-infected at first testing in STF districts compared to the national average (4.1 %) (*p* = 0.4). Twenty-seven percent (n/N = 608/2,226) of HIV-exposed infants had both a date of birth and DNA PCR test date documented. The median age at the time of HIV-1 DNA PCR testing for these 608 infants was 83 days (IQR: 48–166). On average, infants found to be HIV-1 positive underwent first DNA PCR testing at an older age (median: 162 days, IQR: 63–221) than infants testing negative (median: 82 days, IQR: 48–164) (*p* = 0.05). Among HIV-exposed infants with complete EID documentation (n = 608), median age at first DNA PCR testing decreased significantly (*p* <0.001) from 112 days (IQR: 57–198) in year 2 to 76 days (IQR: 46–152) in year 3.

Sufficient documentation was available for 560 infants to determine the approximate average HIV-1 DNA PCR test result turnaround time. For this subgroup, median turnaround time was 49 days (IQR: 32–78 days). We observed no significant difference in approximate turnaround time by HIV-1 DNA PCR test result status (HIV-1 positive: 62 days; HIV-1 negative: 49 days, *p* = 0.3).

## Discussion

We report high facility-level utilization of HTC, ART, and infant nevirapine prophylaxis, and moderate EID services uptake in five Malawi districts supported by STF interventions. STF interventions enhanced the standard of care offered by the national Option B+ program, addressed some health sector needs along the antenatal and early postnatal PMTCT cascade, and contributed to a low proportion of HIV-exposed infants found HIV-1 positive at first DNA PCR testing. Performance on national facility-level PMTCT indicators in STF districts improved by project end, and compared favorably with national averages.

Previous reports of PMTCT programs in Malawi and elsewhere in SSA—prior to Option B + —highlighted several service delivery gaps in the PMTCT cascade, including low uptake of HTC, suboptimal provision of ART to qualifying HIV-infected pregnant women, and inadequate HIV testing among HIV-exposed infants [13, 45–48]. In a recent meta-analysis from SSA, only 70 % of pregnant women received some form of antiretroviral prophylaxis and 40 % of eligible women did not initiate ART [45]. Historically, women have also faced challenges accessing HIV care for their HIV-exposed infants [12, 49]. In Malawi, for example, in a cohort of 14,669 HIV-infected pregnant women and their HIV-exposed infants seeking care in Lilongwe from 2004 to 2008, only 54 % of infants underwent HIV DNA PCR testing, and 14 % of these infants were found to be HIV-infected [12].

In the context of these challenges, we achieved favorable outcomes compared to both national averages and baseline outcome indicators in STF districts. Several reasons may explain the results observed. First, the public health approach taken by the MoH Option B+ program in improving access to maternal ART and infant nevirapine prophylaxis, led to an increased number of women and infants receiving an efficacious PMTCT regimen in our catchment area [14, 50]. Second, the application of basic QI methodology empowered health workers to identify locally relevant strategies to optimize facility-level processes for HTC, ART, and EID service delivery. For example, the establishment of EID “focal persons” responsible for overseeing HIV-exposed infant enrollment and DNA PCR testing in each clinic—an idea first generated from a collaborative QI exercise at Dowa District Hospital and later implemented in all STF districts—may help explain the increased uptake and reduction in median age at first DNA PCR testing observed among HIV-exposed infants over the project period. Third, health worker training and intensive mentorship provided by STF on PMTCT service delivery, including Option B+, may have contributed to more guideline-adherent care and rapid dissemination of best practices. Mounting evidence suggests that longitudinal, structured clinical mentorship contributes to improvements in health-system performance indicators and clinical outcomes, particularly for maternal and child health [51–53]. Fourth, the high uptake of HTC observed may be explained by implementation of opt-out HTC, couples’ HTC, and male partner involvement strategies in ANC clinics. Use of opt-out HTC has been shown to be an effective strategy for increasing HTC utilization in resource-limited settings [18, 54, 55]. Similarly, couples’ HTC and male partner involvement strategies have been associated with beneficial clinical outcomes and health behaviors in SSA, including increased care seeking behavior among HIV-infected women, improved outcomes for HIV-exposed infants, and greater opportunities for linkage to care for HIV-infected men who may be less likely to access HIV services otherwise [33, 56–58]. Fifth, psychosocial support groups for HIV-infected pregnant women may have partially reduced barriers to PMTCT services by ameliorating HIV-associated stigma and encouraging uptake of HTC, ART, and EID services [59]. Lastly, investments in public-sector EID laboratory and ancillary systems, and direct provision of infant HIV-1 DNA PCR testing by STF during EID scale-up may have contributed to the moderate uptake of testing seen among HIV-exposed infants.

We observed an increase in the proportion of HIV-exposed infants receiving DNA PCR testing and earlier uptake of EID testing services over time. However, ensuring high uptake of early HIV-1 testing for all exposed infants in our project area was challenging. In a subgroup of infants with complete documentation, we observed that the median age at first DNA PCR testing was approximately 12 weeks, nearly 6 weeks later than the age recommended by national guidelines. We attribute this to persistent gaps in health worker EID training, DBS sample collection, exposed-infant registration, and exposure screening among infants born at home who later present for routine health services. Infants found to be HIV-infected received DNA PCR testing even later, at an average of almost 6 months of age. While this result may simply reflect the increased HIV-acquisition risk that comes with prolonged breastfeeding duration, the presence of important concomitant barriers to health services utilization cannot be excluded in this sub-group. Even after HIV-exposed infants access EID testing services, health system inefficiencies create delays in providing critical test results to patients and front-line health workers. Among HIV-exposed infants with complete records, the time from DNA PCR testing to result notification for families and healthcare providers was 49 days. Resource limitations and logistical barriers within district-level laboratory and ancillary systems likely contributed to the delays experienced by end-users in receiving timely DNA PCR test results. To address these issues, renewed focus is required to fill persistent gaps in EID services by ensuring an uninterrupted supply of EID laboratory reagents, moving away from paper-based DNA PCR test result notification systems to SMS and web-based platforms, and enhancing community-facility linkages to improve post-natal mother-infant pair follow-up and retention in care. Indeed, scale up and further evaluation of promising community-facility linkage strategies are urgently needed to overcome pervasive challenges with client transport and other structural barriers to care that contribute to delayed service utilization and mother and infant loss to follow-up in Option B+ programs in SSA [8, 10, 60]. The Malawi MoH and implementing partners are committing new resources and evaluating novel strategies—including community health worker support, motorcycle-rider laboratory couriers, POC nucleic acid-based HIV testing, and active tracing of HIV-infected mothers and exposed infants who have fallen out of care—to improve the proportion of exposed infants receiving HIV-1 testing by 2 months of age such that all HIV-infected infants access timely ART [61, 62].

While we demonstrated that facility-level PMTCT service utilization improved over time in our catchment area, and outperformed some important national PMTCT indicators by the end of STF, other factors in addition to STF interventions may explain the results we observed. Such factors include health system strengthening investments and/or PMTCT activities conducted by other implementing partners and non-governmental organizations (NGOs) in STF districts. Notably, strong partnerships between MoH and implementing partners also existed in other regions of the country, so the comparisons we made to national indicators were to national Option B+ program outcomes that were also NGO-supported. In addition to the possible influence of external factors, our study had several other limitations. First, we were unable to estimate PMTCT service utilization among HIV-infected women who did not have at least one ANC clinic visit or who did not undergo facility-based HTC. An estimated 8 % of pregnant women did not have an ANC clinic visit during the project period, and over one-third of ANC attendees did not undergo HTC in years 1 and 2 largely due to HIV test kit shortages pervasive in Malawi at the time [35, 63, 64]. This may have led our facility-level indicators to overestimate true service utilization in the population of HIV-infected pregnant women and exposed infants in our catchment area. Second, while we provided HIV-1 DNA PCR testing to over 2,000 HIV-exposed infants, this represented just 20 % of the total number of infants undergoing testing in our catchment area during the project period, limiting the generalizability of our findings about STF impact on early MTCT. Third, the cross-sectional nature of our facility-level indicators prevented us from drawing inferences about the effects of STF interventions on individual-level maternal and infant outcomes over time, including the proportion of HIV-infected infants linked into HIV care and treatment. To address these issues, additional studies in central Malawi are ongoing using linked, mother and infant cohort data to understand the patient-level factors associated with vertical HIV transmission under routine operational conditions, and to estimate the adjusted individual-level risk of loss to follow up for Option B+ clients and newly diagnosed HIV-infected infants in STF-supported districts.

## Conclusions

We describe STF interventions, integrated within the national Option B+ program, that addressed gaps along the antenatal and early postnatal PMTCT cascade and that strengthened PMTCT services for over half a million pregnant women in central Malawi. We note high facility-level utilization of HTC, ART, and infant nevirapine prophylaxis, and moderate uptake of EID services in STF districts, comparing favorably to national averages and differing from prior reports of inadequate service delivery and low PMTCT service uptake in SSA. We speculate that by enhancing the national Option B+ program, STF interventions contributed to the lower proportion of HIV-exposed infants found HIV-1 positive at first DNA PCR testing in STF districts compared to the national average. Several STF interventions, including health worker mentorship, facility-level QI, and couples’ HTC, have since influenced or been adopted by the national Option B+ program to strengthen the PMTCT cascade throughout Malawi, suggesting the scalability and sustainability of our approach. Lastly, we report findings from an individual-level analysis of infant DNA PCR test results in which we observed progressive reductions in the age at first testing over the STF project period, despite ongoing challenges of prolonged result turnaround times and delayed first DNA PCR testing. To address these challenges and to build upon the early success of Option B+ in Malawi, governmental and non-governmental partners should reaffirm their commitment to improving EID services and continue to invest in ameliorating gaps along the PMTCT cascade.
